# Personalized signaling models for personalized treatments

**DOI:** 10.15252/msb.20199042

**Published:** 2020-01-09

**Authors:** Julio Saez‐Rodriguez, Nils Blüthgen

**Affiliations:** ^1^ Faculty of Medicine Heidelberg University and Heidelberg University Hospital Institute for Computational Biomedicine Bioquant Heidelberg Germany; ^2^ Faculty of Medicine Joint Research Centre for Computational Biomedicine (JRC‐COMBINE) RWTH Aachen University Aachen Germany; ^3^ Institute of Pathology Charité ‐ Universitätsmedizin Berlin Berlin Germany; ^4^ IRI Life Sciences Humboldt Universität zu Berlin Berlin Germany

**Keywords:** Cancer, Computational Biology, Signal Transduction

## Abstract

Dynamic mechanistic models, that is, those that can simulate behavior over time courses, are a cornerstone of molecular systems biology. They are being used to model molecular mechanisms with varying degrees of granularity—from elementary reactions to causal links—and to describe these systems by various dynamic mathematical frameworks, such as Boolean networks or systems of differential equations. The models can be based exclusively on experimental data, or on prior knowledge of the underlying biological processes. The latter are typically generic, but can be adapted to a certain context, such as a particular cell type, after training with context‐specific data. Dynamic mechanistic models that are based on biological knowledge have great potential for modeling specific systems, because they require less data for training to provide biological insight in particular into causal mechanisms, and to extrapolate to scenarios that are outside the conditions they have been trained on.

## Graphical abstract

Dynamic mechanistic models have been broadly applied to analyze signaling (dys‐)regulation in cancer. Patient‐specific dynamic models are promising for understanding the mechanisms underlying disease and drug responses and for developing personalised therapies.

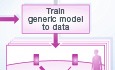

Such models have been broadly applied to study signal transduction in mammalian cells and to analyze signaling (dys‐)regulation in disease, in particular in cancer. By integrating the mode of action of drugs with causal relationships between molecular components inferred from dynamical data, these models can be particularly useful for understanding and even predicting the effects of targeted therapies. In addition, they can provide insights into the adaptive behavior and rewiring of signaling processes in response to drug treatment. Importantly, mechanistic models are also used to infer information about cellular processes and molecules that cannot be directly measured: so‐called hidden (or latent) variables. Additionally, mechanistic models of signaling can be expanded to model downstream processes and phenotypic responses, such as cell proliferation and death, which are particularly relevant for studying drug efficacy in the context of cancer. However, it is often not well understood how exactly these downstream processes are connected to “upstream” signaling events and which signaling features cause cellular decisions. In such cases, the models are semi‐mechanistic or based on statistical inference.

A key challenge to efficiently using dynamic mechanistic models for personalized treatments or stratifying patients is making them patient‐specific (Fig 1). Our understanding of signaling is often based on experiments performed in defined *in vitro* contexts that do not necessarily reflect the cellular context of interest, that is, of a patient sample. The fact that the information in databases is mainly agnostic to cell types and contexts makes deriving cell‐type‐specific and patient‐specific models a complex challenge.

**Figure 1:**
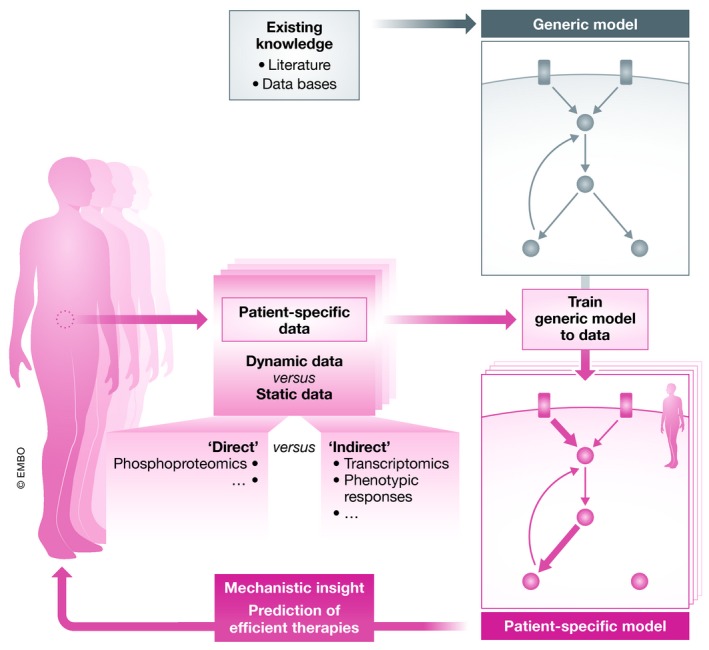
Schematic of the cycle to generate patient‐specific dynamic models A generic model, not tailored to a patient or cell line, can be built from existing knowledge. This model can be trained to data to build a patient‐specific model (alternatively, the model can be purely generated from the data). The model can then be used to generate predictions of therapies on the patient.

One strategy for parameterizing models for a specific cell type or patient is using data obtained for the subject of interest. Arguably, the most informative data source for calibrating a model is perturbation experiments, which measure the system's response to a stimulation or inhibition of one or multiple nodes in the network. Such data contain information about the dynamics (by monitoring evolution over time) and causality (by observing the effect of defined alterations on other network components). This type of data can be obtained relatively easily if the material is abundant, for instance when performing *in vitro* experiments with specific cell lines.

Previous studies have shown that data from cell lines from the same tumor type can be used to build models that reflect the heterogeneity of signaling network behavior between different tumor subtypes. For example, ordinary differential equation (ODE) models parameterized with data from cell lines have been used to understand cellular responses to therapy and to optimize combinatorial therapies in melanoma cells (Korkut *et al*, 2015). Qualitative networks, an extension of Boolean models (Silverbush *et al*, 2017) calibrated with genetic and perturbation data on four leukemia cells, have provided similar insights. Cell‐line‐specific logic‐ODE models trained on perturbation data from a panel of 14 colorectal cancer cell lines revealed parameters that correlate well with drug sensitivity and that can be used to choose combination therapies (Eduati *et al*, 2017). More coarse‐grained models based on modular response analysis that was trained on perturbation data from a small panel of colon cancer cell lines uncovered the ubiquitous resistance mechanism of feedback regulation in these cells which could be overcome by combinatorial treatments; this was validated in a xenograft model (Klinger *et al*, 2013). These examples demonstrate that models based on data from cultured cells can be powerful tools to optimize therapies for broader classes of tumors. Nevertheless, using data from cell lines presents certain limitations, since cell lines generally have altered signaling compared with primary cells, and do not reflect a specific patient context. Even more important, they do not reflect the intra‐patient heterogeneity that results either from genetic or from cellular heterogeneity owing to cell hierarchies of differentiation within tissues.

In order to accurately reflect the patient‐specific context, we would ideally need to generate (perturbation) data directly from patient‐derived material. However, this is not possible for all tissues and disease contexts. While blood can be obtained relatively easily in sufficient amounts, material from solid tissues is typically limited. If the tumor is surgically removed as part of the treatment, there is the possibility to obtain some tumor material. In other pathologies, either no material is removed, or only a small amount is obtained as a biopsy for diagnosis. Therefore, experimental technologies that perform perturbation screenings with large throughput from small amounts of material can be really helpful. Microfluidic platforms to analyze low amounts of patient material are promising technologies in this regard and have already been used to generate data for patient‐specific models (Eduati *et al*, 2018). However, they also have limitations, including the number of available readouts and the fact that by suspending the cells the tissue structure and interactions are lost.

These *ex vivo* approaches are still in their infancy, and as a result, such data are not yet broadly available. While we expect them to become increasingly popular, alternative strategies that use more common and easier to obtain data will be important, in particular in the short term. An alternative is using basal data, e.g., quantitative or qualitative data from a patient sample to contextualize the model. This is fundamentally different from model training with perturbation data. Perturbation data are directly usable for training, since the responses predicted by the model after specific stimulations or inhibitions can be compared to the perturbation data. In contrast, basal data can only be used to modify specific model parameters, such as the concentration of molecular components or changes in the model's structure. For instance, specific mutations in a patient can render a protein dysfunctional, and this information can be encoded in the model by removing certain nodes (proteins) or edges (interactions). Proteomic data, or transcriptomic data as a proxy, can inform on protein levels and can be used to reparametrize and personalize cell‐line‐derived models (Fey *et al*, 2015; Barrette *et al*, 2018).

However, while protein levels can serve as an indication of protein activity and consequently of signal transduction, additional processes, including post‐translational modifications (e.g., phosphorylation) or subcellular localization, further control protein activity. It is therefore not surprising that when using basal data focused on protein amounts, ODE‐type (Fröhlich *et al*, 2018) or Boolean models (Béal *et al*, 2019) show only modest performance in predicting drug responses. Alternatively, pathway signatures extracted from transcriptome data might be a good proxy to assess pathway activity, in particular using the changes in expression induced by pathways, that is, its “footprint” on gene expression. Such signatures can be used to infer upstream‐activated pathways by means of reverse‐causal reasoning tools.

Overall, it is important to study the process of interest using material that most closely resembles the patient context. For many tissues and tumor types, 3D culture systems, also known as organoid cultures, can be used to grow and expand tissue samples directly from patient material. These culture systems also allow generating heterogeneous cell populations, which, in several cases, resemble cell hierarchies in the underlying tissue. The combination of 3D culture systems with single‐cell analysis, such as mass cytometry and scRNA‐seq, allows generating perturbation‐response data that capture the heterogeneous cellular responses within a heterogeneous tissue (Brandt *et al*, 2019). These systems can even capture heterogeneity within one patient and are a suitable experimental tool for deriving data for patient‐specific models. Organoids have been extensively used for intestinal tissues, but are rapidly becoming available for other tissues and systems. By way of example, they allow growing complex structures such as neuronal tissues from patient materials in conjunction with induced pluripotent stem cells and advanced differentiation protocols. Importantly, genome editing methods can also be applied to organoids, which create the possibility to examine the effects of patient‐specific mutations on signaling within the context of specific cell types and cell hierarchies for establishing patient‐specific models. To make sure that the organoids truly represent the tissue of the patient, it is crucial to systematically compare their cell‐type composition and expression profiles within cells between organoids and directly sampled patient material. Such comparisons can be performed with relatively good accuracy using single‐cell transcriptomics.

We expect that technological developments including patient‐derived organoids, CRISPR genome editing, microfluidic platforms, and single‐cell technologies will soon allow generating rich perturbation datasets. These datasets can in turn be used to generate increasingly accurate patient‐specific mechanistic models. Additionally, organ‐on‐chip technologies can be used to model experimentally more complex interactions between cell types and even organs, and will also aid the generation of more complex computational models. Such models will likely provide insights into how drugs rewire signaling and how drug resistance occurs. Nevertheless, it remains open whether these models, which incorporate our mechanistic understanding derived from decades of research on cellular signaling, are clinically predictive or will be eventually outcompeted by pure statistical machine‐learning methods. Clearly defined benchmarks such as the DREAM challenges, where researchers can test their methods and compare their performance, will be essential to tackle this question before conducting clinical studies that use models for making decision on therapies. Overall, the technologies and approaches described above seem promising for ultimately leading to the wider use of mechanistic models for understanding the mechanisms underlying disease and drug responses and for developing truly personalized therapies.

## Conflict of interest

The authors declare that they have no conflict of interest.

## References

[msb199042-bib-0001] Barrette AM , Bouhaddou M , Birtwistle MR (2018) Integrating transcriptomic data with mechanistic systems pharmacology models for virtual drug combination trials. ACS Chem Neurosci 9: 118–129 28950062 10.1021/acschemneuro.7b00197 PMC5771884

[msb199042-bib-0002] Béal J , Montagud A , Traynard P , Barillot E , Calzone L (2019) Personalization of logical models with multi‐omics data allows clinical stratification of patients. Front Physiol 9: 1965 30733688 10.3389/fphys.2018.01965 PMC6353844

[msb199042-bib-0003] Brandt R , Sell T , Lüthen M , Uhlitz F , Klinger B , Riemer P , Giesecke‐Thiel C , Schulze S , El‐Shimy IA , Kunkel D *et al* (2019) Cell type‐dependent differential activation of ERK by oncogenic KRAS in colon cancer and intestinal epithelium. Nat Commun 10: 2919 31266962 10.1038/s41467-019-10954-y PMC6606648

[msb199042-bib-0004] Eduati F , Doldàn‐Martelli V , Klinger B , Cokelaer T , Sieber A , Kogera F , Dorel M , Garnett MJ , Blüthgen N , Saez‐Rodriguez J (2017) Drug resistance mechanisms in colorectal cancer dissected with cell type‐specific dynamic logic models. Cancer Res 77: 3364–3375 28381545 10.1158/0008-5472.CAN-17-0078 PMC6433282

[msb199042-bib-0005] Eduati F , Jaaks P , Merten CA , Garnett MJ , Saez‐Rodriguez J (2018) Patient‐specific logic models of signaling pathways from screenings on cancer biopsies to prioritize personalized combination therapies. bioRxiv 10.1101/422998 PMC7029724 32073727

[msb199042-bib-0006] Fey D , Halasz M , Dreidax D , Kennedy SP , Hastings JF , Rauch N , Munoz AG , Pilkington R , Fischer M , Westermann F *et al* (2015) Signaling pathway models as biomarkers: patient‐specific simulations of JNK activity predict the survival of neuroblastoma patients. Sci Signal 8: ra130 26696630 10.1126/scisignal.aab0990

[msb199042-bib-0007] Fröhlich F , Kessler T , Weindl D , Shadrin A , Schmiester L , Hache H , Muradyan A , Schütte M , Lim J‐H , Heinig M *et al* (2018) Efficient parameter estimation enables the prediction of drug response using a mechanistic pan‐cancer pathway model. Cell Syst 7: 567–579.e6 30503647 10.1016/j.cels.2018.10.013

[msb199042-bib-0008] Klinger B , Sieber A , Fritsche‐Guenther R , Witzel F , Berry L , Schumacher D , Yan Y , Durek P , Merchant M , Schäfer R *et al* (2013) Network quantification of EGFR signaling unveils potential for targeted combination therapy. Mol Syst Biol 9: 673 23752269 10.1038/msb.2013.29 PMC3964313

[msb199042-bib-0009] Korkut A , Wang W , Demir E , Aksoy BA , Jing X , Molinelli EJ , Babur Ö , Bemis DL , Sumer SO , Solit DB *et al* (2015) Perturbation biology nominates upstream–downstream drug combinations in RAF inhibitor resistant melanoma cells. Elife 4: e04640 10.7554/eLife.04640 PMC4539601 26284497

[msb199042-bib-0010] Silverbush D , Grosskurth S , Wang D , Powell F , Gottgens B , Dry J , Fisher J (2017) Cell‐specific computational modeling of the PIM pathway in acute myeloid leukemia. Cancer Res 77: 827–838 27965317 10.1158/0008-5472.CAN-16-1578

